# P26 Felty’s syndrome as the first presentation of rheumatoid arthritis

**DOI:** 10.1093/rap/rkad070.047

**Published:** 2023-09-27

**Authors:** Fatima Salih, Wiranthi Gunasekera

**Affiliations:** North West Anglia NHS Foundation Trust, Peterborough, United Kingdom; North West Anglia NHS Foundation Trust, Peterborough, United Kingdom

## Abstract

**Introduction:**

Felty's syndrome (FS) is a rare, autoimmune disease occurring in < 1% of patients with rheumatoid arthritis (RA) and comprising a triad of persistent idiopathic neutropenia, splenomegaly and RA. In some affected individuals, FS may develop when the symptoms and signs of RA have subsided or are absent. In this case, FS may remain undiagnosed. In rare instances, the development of FS may precede the development of the clinical manifestation of RA.

**Case description:**

A 73-year-old Caucasian gentleman presented in October 2017 with pancytopenia and splenomegaly. He was investigated by the haematology team with multiple bone marrow biopsies showing no underlying haematological causes for his pancytopenia.

He was also investigated by the hepatologists for the possibility of liver cirrhosis as he had a heavy alcohol intake. His liver ultrasound showed fatty liver with splenomegaly, his fibro-scan showed fibrosis with no evidence of cirrhosis and his OGD showed no oesophageal varies. At this point, he was treated with blood transfusions, weekly erythropoietin and filgrastim as needed under the haematologists.

In October 2022, he was admitted with febrile neutropenia and he gave a 20-year history of palindromic rheumatism with no evidence of synovitis or previous DMARD treatment. His rheumatoid factor (RF) was >120 iu/ml, anti-cyclic citrullinated peptide (ACCPA) was >340 u/ml and ANA by CIA was weakly positive (20.6 CU) with Hep-2 IFF being homogenous. ENA and dsDNA were negative. His hand X-ray showed evidence of osteoarthritis with no erosion.

He subsequently had multiple admissions due to infections. CT chest, abdomen, and pelvis showed splenomegaly with portal vein dilatation suggestive of portal hypertension, normal liver appearance, and left lower lung bronchiectasis. Liver biopsy showed non-specific portal and sinusoidal inflammation, mild fibrosis, and architectural features suggestive of nodular regenerative hyperplasia indicative of FS. Furthermore, his HLA-DRB1*04 was positive. Together, these factors allowed us to diagnose FS.

He was started on a tapered course of prednisolone, 30mg daily decreased by 10mg every two weeks, and methotrexate (10mg orally weekly) with careful blood monitoring. Two weeks later, he developed a mild rise in the ALT and alkaline phosphatase. Therefore, the methotrexate dose was reduced to 5mg weekly. Four weeks later, he reported feeling much better, with improvement in all his cell counts and liver function.

**Discussion:**

While persistent idiopathic neutropenia and splenomegaly (present in 90% of FS patients) are salient features of FS, patients can also present with other extra-articular manifestations such as rheumatoid nodules (74%), hepatomegaly (68%), lymphadenopathy (42%), Sjögren's syndrome (48%), pulmonary fibrosis (50%), pleuritis (22%), peripheral neuropathy (14%) and leg ulcers (16%). Systemic symptoms include fever and weight loss. They also present with idiopathic non-cirrhotic portal hypertension, which can lead to variceal bleeding.

The exact pathophysiology of FS is unclear, making the diagnosis and treatment of FS very challenging. A large multi-central cohort study found that FS is associated with HLA-DRB1*0401 and, in two other studies into the pathophysiology of FS, they found that 73% of patients with FS had IgG Anti-G-CSF and autoantibodies that bind to deaminated histones and neutrophil extracellular chromatin traps (NETs), leading to neutrophil sequestration. Therefore, treatment aims to control the underline RA, improve the neutrophil count, and prevent infection.

The first challenge, in this case, was to confirm the diagnosis of FS in the absence of any obvious RA joint involvement and a history of heavy alcohol intake. Following an extensive investigation, we discussed his case at the Rare Disease MDT meeting and liaised with the haematology, hepatology, and histopathology teams to exclude the other differential diagnoses.

The clues to the diagnosis of FS were the strongly positive RF and ACCPA, the positive HLA-DRB1*04, the liver biopsy, and the exclusion of other causes of neutropenia.

The second challenge in this case was to safely start methotrexate, given his mild liver fibrosis and recurrent infections. With close blood test monitoring and follow-up, he achieved improvement in his cell count (WBC from 0.8 to 1.1x10 9/L, HB from 86 to 104 g/L, platelet from 70 to 91X109/L) with stable liver function.

**Key learning points:**

Rheumatoid arthritis is a multisystem disease with variable presentations. Rarely, FS can occur in the absence of clinical manifestations of the erosive joint involvement of RA. In this situation, careful, thorough investigation to exclude other causes of neutropenia and prove the diagnosis of FS is imperative to justify immunosuppressive treatment. A multidisciplinary team working across specialties was pivotal in the safe diagnosis and treatment of this gentleman.

